# Therapeutic approach to "downhill" esophageal varices bleeding due to superior vena cava syndrome in Behcet's disease: a case report

**DOI:** 10.1186/1471-230X-6-43

**Published:** 2006-12-27

**Authors:** Hamid Tavakkoli, Mehrnaz Asadi, Mahshid Haghighi, Abbas Esmaeili

**Affiliations:** 1 Alzahra Hospital, Isfahan University of Medical Sciences, Isfahan, Iran; 2 Poursina Hakim Research Institute, Isfahan, Iran

## Abstract

**Background:**

One of the rare presentations of superior vena cava syndrome is bleeding of "downhill" esophageal varices (DEV) and different approaches have been used to control it. This is a case report whose DEV was eradicated by band ligation for the first time.

**Case presentation:**

We report a 42-year-old man who is a known case of Behcet's disease. The patient's first presentation was superior vena cava syndrome due to thrombosis followed by bipolar ulcers and arthralgia. He received warfarin, prednisolone and azathioprine. The clinical course of the patient was complicated by one episode of hematemesis without abdominal pain when the patient's PT was in therapeutic range. After resuscitation and correction of PT with fresh frozen plasma transfusion, upper gastrointestinal endoscopy was done. Prominent varices were seen in the upper third of the esophagus, tapering to the middle part without acute bleeding. Stomach and duodenum were normal. Color ultrasonography evaluation of the portal, hepatic and splenic veins was negative for thrombosis. Band ligation was done and the patient's bleeding did not recur.

**Conclusion:**

Band ligation is a safe and effective method for controlling DEV bleeding in patients with uncorrectable underlying disorders.

## Background

"Downhill" esophageal varices (DEV) which develop in the upper third of the esophagus are less common than distal esophageal varices, i.e. the uphill type, which is usually produced by portal hypertension [1,2]. DEV serve as collateral branches directing blood flow "downwards", either to bypass superior vena cava [SVC] obstruction via azygus vein, or to drain the superior systemic system to the portal vein when both the SVC and the azygus vein are obstructed [3]. Predominant factors involved in the determination of the downward extension of varices along the esophagus are the level of SVC obstruction and its duration [2,4]. The more distal the obstruction of SVC and the slower the process of occlusion, the higher the possibility of DEV developing. With obstruction of the SVC and the azygus vein, venous blood flow from the cranium and upper extremities may flow through the inferior thyroid veins and mediastinal collaterals into esophageal veins and then into the coronary vein to the portal vein, hepatic vein and inferior vena cava to the heart, resulting in "downhill" varices [4,5].

"Downhill" varices are mostly due to SVC syndrome secondary to mass effects including lung cancers [6-9], intrathoracic goiter [3,10-12], mediastinal lymphoma [13], thyroid carcinoma [14,15], thymoma [2,16], mediastinal lymphadenopathy secondary to different head, and neck cancers such as carcinoma of the tongue [7]. Less frequent etiologies of DEV are Behcet's disease [17-20], systemic venulitis [21], thyroid disease or a history of thyroid surgery [10,22], fibrosing mediastinitis [23], as a complication of upper extremity hemodialysis access [24], Castleman's disease (angiofollicular lymph node hyperplasia) [1], muscular constriction of abnormal extensions of posterior hypopharyngeal veins, and venous obstruction as a rare late complication after correction of congenital heart defect [25], and interestingly, one report of liver cirrhosis [26]. Intramural esophageal hematoma (pseudo-DEV) should also be considered in the differential diagnosis [27].

## Case Presentation

**Figure 1:**
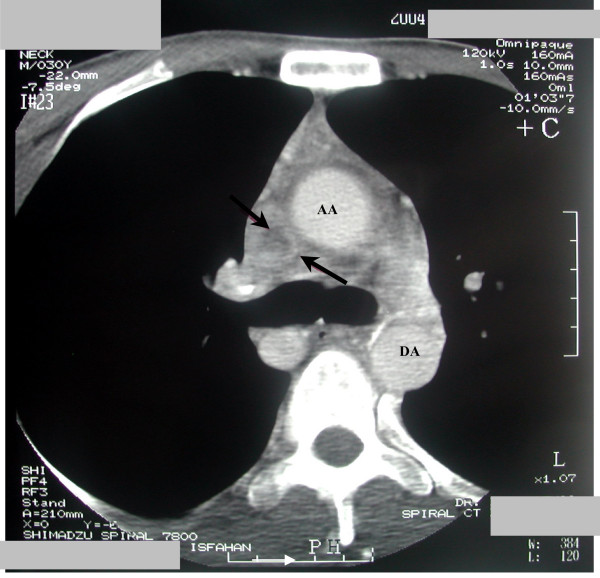
Spiral CT scan at the level of carina. SVC partial thrombosis (black arrows). AA: Ascending Aorta. DA: Descending Aorta

**Figure 2:**
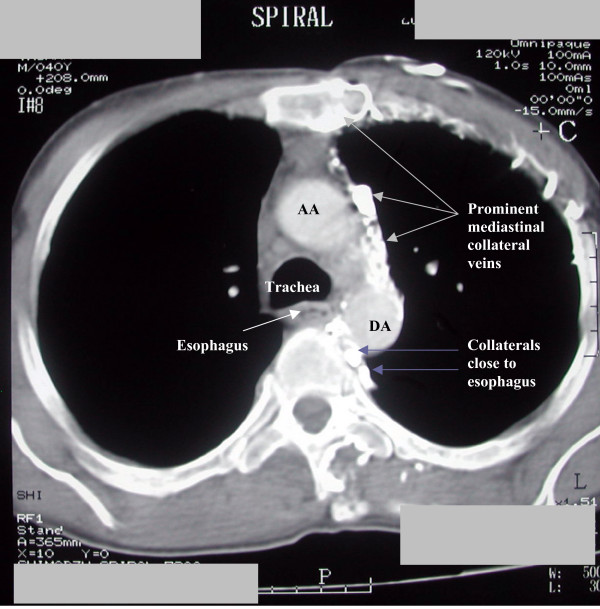
Spiral CT of the thorax at the level of aortopulmonary window.

A 42-year-old man presented with sudden-onset hematemesis. Everything had started a year before, when he had developed shortness of breath along with a plethoric face. The patient was admitted to the Surgery Department and was diagnosed with SVC thrombosis by means of Doppler ultrasonography and spiral computed tomography (CT) scan of chest (figure 1). The subject underwent thoracotomy out of suspicion of mediastinal mass effect on SVC, detected on thoracic spiral CT scan to be a thymoma or a thymus-associated mass. In the operation, no discrete mass was found and the thymus gland was removed and sent for pathologic review. No pathologic finding was detected and the gland had normal histological appearance. The patient received anticoagulant therapy in the form of warfarin 5 mg/day for 9 months. Then, he developed arthralgia of knee, wrist, elbow and ankles but no documented arthritis. He was examined by a rheumatologist when he was discovered to have positive pathergy test (positive cutaneous hypersensitivity reaction to intradermal injection of saline) and an oral aphthous ulcer. The history of genital ulcer was also positive in the patient. The diagnosis of Behcet's disease was made and the patient was treated with prednisolone tablets, 15 mg/day, and azathioprine, 100 mg/day; warfarin was also continued. A follow-up CT scan showed prominent mediastinal collateral veins close to the esophagus (figure 2).

Two weeks later, the patient presented with sudden onset of hematemesis and was admitted to the Emergency Room. He did not mention any past history of upper gastrointestinal bleeding. Review of systems was not contributory. The patient was conscious and agitated. His blood pressure was 110/70 mmHg, but he had orthostatic hypotension with resting heart rate of 104/min. The patient had plethoric face and engorgement of jugular vein. Conjunctivae were pale and sclerae were anicteric. Pemberton's sign was detected; i.e. within 30 seconds after raised both arms simultaneously (Pemberton's maneuver), marked facial plethora (Pemberton's sign) developed, indicating compression of the jugular vein. Abdomen was soft with no tenderness, organomegaly or shifting dullness. Nervous system, musculoskeletal system, joints and the peripheral vascular system were all normal. A nasogastric tube was inserted and coffee ground secretions were revealed. Lab data were as follows: WBC: 6400, Hb: 6.5 g/dl, MCV: 97, MCH: 32, Platelet: 241,000, PTT: 40 seconds, PT: 18 seconds, INR: 2.1, albumin: 4 g/dl, ALT: 23, Alkaline Phosphatase: 170 (normal), BUN: 18 mg/dl, Cr: 0.6 mg/dl, Na: 138 meq/dl, K: 4.2 meq/dl.

The patient received 6 fresh frozen plasma (FFP) units and 4 units of packed red cells and his INR was rechecked after FFP infusion, when it was corrected to 1.3.

**Figure 3:**
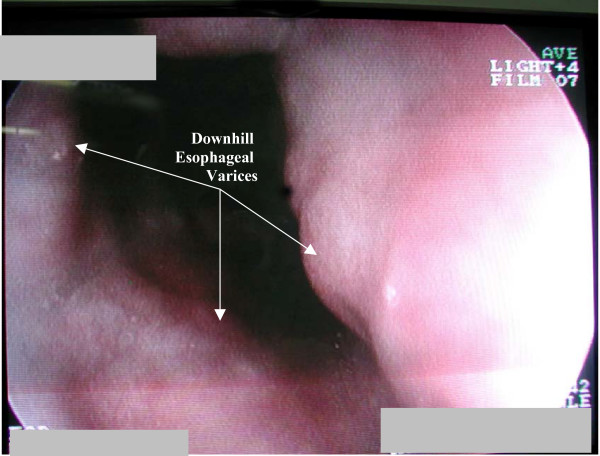
Endoscopic findings on upper third of esophagus.

**Figure 4:**
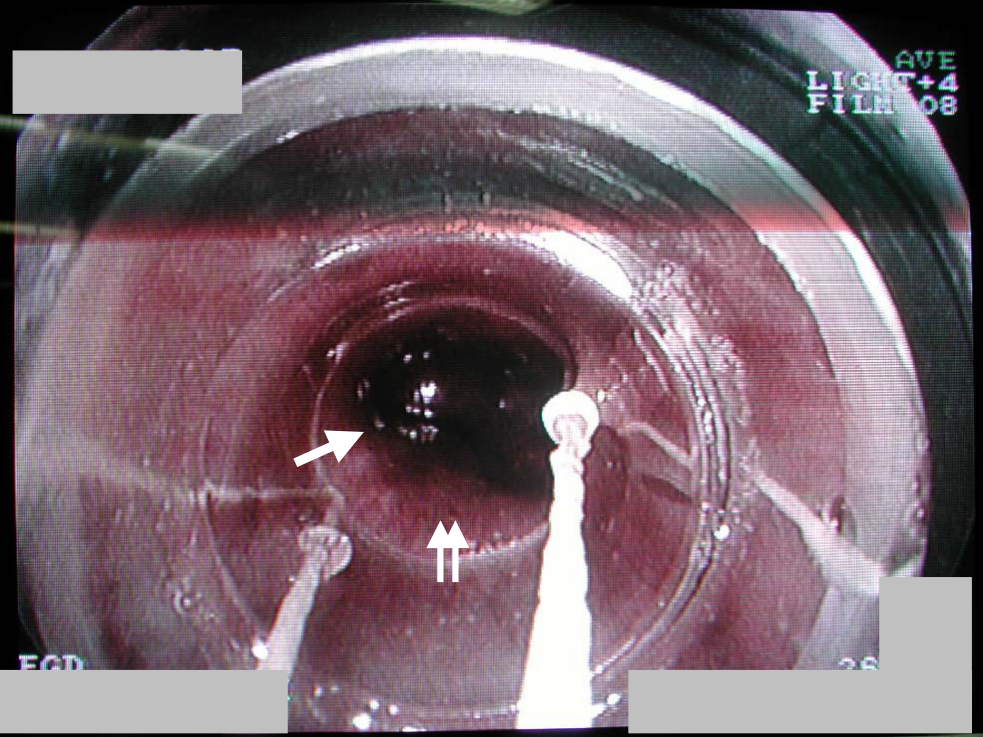
Ligated esophageal varice (single arrow) just below upper esophageal sphincter (double arrows).

Because of underlying vasculitis, the diagnosis of portal vein thrombosis and bleeding due to esophageal varices was considered. The patient received intravenous infusion of octreotide and intravenous infusion of ranitidine. The bleeding stopped and the patient was closely monitored in the Emergency Room. Warfarin and azathioprine were discontinued and intravenous hydrocortisone was started instead of oral prednisolone. The patient underwent upper endoscopy on the day of admission and esophageal varices were found. Gastric and duodenal mucosae were intact. Abdominal ultrasound was normal and no evidence of ascites, splenomegaly and chronic liver disease was found. To rule out portal vein thrombosis as the cause of esophageal varices, Doppler ultrasonography was performed and mesenteric, splenic, portal and hepatic veins were evaluated; no discrete thrombosis was detected. He was not able to perform MR angiography because of his economic status. The patient underwent re-endoscopy by a second endoscopist and red color sign or stigma of recent bleeding plus esophageal varices were found in the upper half of esophagus tapering to the middle part of esophagus (figure 3); the diagnosis of "downhill" esophageal varices was made and band ligation was performed successfully (figure 4). He received oral cyclophosphamide 100 mg/day upon recommendation of rheumatologist. Omeprazole was administered too.

The patient was discharged from hospital the following day without any complication. Two weeks later, the third endoscopy along with the second band ligation was performed. He was discharged without any further episodes of bleeding. After 1 and 6 months, the patient underwent the 4th and 5th endoscopies and "downhill" esophageal varices were found to have been eradicated. Warfarin was initiated for patient and he has been well with no bleeding during the past 16 months. We are going to do surveillance endoscopies in this patient every 1–2 years.

## Discussion

Mediastinal tumor-induced DEV is seen sporadically, but one due to SVC obstruction secondary to Behcet's disease is very rare [20]. Interestingly, SVC syndrome could be the initial symptom in Behcet's disease [18] as in the presented case. The most frequent signs and symptoms were face or neck swelling (82%), upper extremity swelling (68%), dyspnea (66%), cough (50%), and dilated chest vein collaterals (38%). Dyspnea at rest, cough, and chest pain were more frequent in patients with malignancy [28]. One of the unseen manifestations of SVC syndrome often discovered accidentally is DEV and upper gastrointestinal bleeding could be its first presentation. After reviewing approximately 130 cases of DEV, Maton et al. found only 9% to have gastrointestinal hemorrhage, much lower than that in uphill varices [21]. But the bleeding could be life-threatening [21,24].

In some cases, varices disappear following treatment of the underlying etiology (table 1), e.g. treatment of systemic vasculitis by steroids and dapsone [21], chemoradiotherapy or resection of the tumor [1,13] or thyroidectomy in the presence of intrathoracic goiter [10,11,15]. On the other hand, since fatal pulmonary embolism may happen after endoscopic sclerotherapy of "downhill" esophageal varices, cyanoacrylate injection into a varice above the lower third of the esophagus is a high-risk procedure [29]. In the presented case, eradication of upper esophageal varices didn't lead to any other visible variceal development either in hypopharynx or lower esophagus or stomach up to six months after band ligation. Then, band ligation seems to be a safe method of controlling DEV bleeding in patients with uncorrectable underlying disorders. But, further evaluations with large number of patients are warranted before drawing definite conclusions.

## Competing interests

The author(s) declare that they have no competing interests.

## Authors' contributions

HT was the gastroenterologist diagnosed DEV for the first time in this case and did the band ligation. MA was the internal medicine resident involved in patient management, who took a detailed history, recorded laboratory data and follow-up information. MH was the radiologist who also contributed to diagnosis through imaging evaluation. AE, HT, MA and MH all contributed by providing the figures. AE prepared the primary draft of manuscript and finally, all authors contributed in final manuscript preparation.

## Pre-publication history

The pre-publication history for this paper can be accessed here:



## Figures and Tables

**Table 1:** Different therapeutic approaches to DEV.

**Category (reference)**	**Etiology and/or Indication**	**Major Manifestation**	**Therapeutic Approach**	**Results of Treatment**	**Possible Complications of Treatment**
1 [10; 11; 15]	Intrathoracic goiter	Bleeding	Thyroidectomy	Usually complete response	Complications of surgeries
2 [1]	Tumor	Bleeding	Chemoradiotherapy	Partial response	Chemoradiotherapy side effects
3 [13]	Tumor	Bleeding	Surgical Resection	Usually complete response	Complications of surgeries
4 [21]	Systemic vasculitis	Bleeding	Steroids and dapsone	Complete response	Medications side effects
5 [29]	Unspecified disease	Bleeding	Sclerotherapy	Partial or no response	Bleeding, fatal pulmonary embolism
6 [current report]	Behcet's disease	Bleeding	Band ligation	Complete response	Endoscopic band ligation complications
